# The influence of incarceration and Re-entry on the availability of health care organizations in Arkansas

**DOI:** 10.1186/s40352-015-0016-4

**Published:** 2015-02-24

**Authors:** Danielle Wallace, John M Eason, Andrea M Lindsey

**Affiliations:** 1 School of Criminology and Criminal Justice, Arizona State University, 411 N. Central Ave., Room 600, Phoenix, AZ 85004 USA; 2 Department of Sociology, Texas A&M University, 311 Academic Building, College Station, TX 77843 USA; 3 Department of Criminology and Criminal Justice, Florida State University, Eppes Hall 112 S. Copeland Street Tallahassee, Florida, 32306-1273 USA

**Keywords:** Incarceration, Re-entry, Coercive mobility, Health care organizations

## Abstract

**Background:**

Studies show that ex-prisoners often experience more health problems than the general population; unfortunately, these issues follow them upon their release from prison. As such, it is possible re-entry rates signal the need for neighborhood-based health care organizations (HCOs). We ask: are incarceration and re-entry rates associated with the availability of HCOs?.

**Methods:**

MethodsUsing 2008 Central Business Pattern data, 2008 prison admissions and release data, and 2000 and 2010 census data, we test whether prison admission and release rates impact the availability of HCOs net of neighborhood characteristics in Arkansas using Logit-Poisson hurdle models with county fixed effects.

**Results:**

We find that the incarceration and re-entry rates – together known as coercive mobility -- are related to whether a neighborhood has one or more HCOs, but not to the number of HCOs in a neighborhood.

**Conclusion:**

Future public policies should aim to locate health care organizations in areas where there is significant churning of individuals in and out of prison.

**Electronic supplementary material:**

The online version of this article (doi:10.1186/s40352-015-0016-4) contains supplementary material, which is available to authorized users.

## Background

Approximately 800,000 people are released from prison each year (Carson and Sabol, 2012), and many of these individuals return to their communities with health problems. Due to prison overcrowding, close quarters, and high levels of contact, prisoners generally suffer from high rates of infectious disease (Massoglia, 2008). Travis et al. (2001) shows that 15% of individuals with HIV have passed through correctional facilities; similarly, 40% of all people with hepatitis C had contact with correctional facilitates (Travis et al., 2001). Chronic diseases and mental health issues are more prevalent among prisoners too, with the incidence of mental illness among prisoners being 2 to 3 times higher than that of the general population (Freudenberg, 2001), and chronic conditions occurring in nearly 80% of all male prisoners (Mallik-Kane and Visher, 2008). Moreover, former prisoners have higher mortality rates than the general population. Female ex-prisoners, for example, have higher rates of death than male ex-prisoners, though both male and female ex-prisoners have mortality rates that are, on average, 3.5 times higher than that of the general population (Binswanger et al., 2007). All said, it is not surprising that incarceration is linked to deleterious effects on individual and population health (Auchincloss et al., 2008; Spaulding et al., 2011; Thomas, 2006; Wildeman, 2011; Wildeman, 2012; for an exception, see Akers et al., 2012; Potter, 2007).

Thus, when prisoners re-enter communities, are just as likely in need of health care as when they enter prison. The poor health of current and former prisoners is partially due to their experiences prior to incarceration (Akers et al., 2012; Spaulding et al., 2011). Prisoners likely come from impoverished areas and backgrounds where they had limited access to health care (Hipp et al., 2009; Kirk, 2009); as such, jails and prisons tend to inherit the health problems of those individuals (Potter, 2007). When exiting prison, health care organizations become more important to ex-prisoners because obtaining health services, such as substance abuse or mental health treatment, are often conditions of parole or probation (Kubrin and Stewart, 2006). Indeed, one study demonstrates that prisoners often return to the community with substance abuse issues that have not be dealt with while incarcerated (Chamberlain, 2012). As such, limited access to health care resources may be a barrier to successful reentry (Petersilia, 2003; Travis, 2005).

Unfortunately, ex-prisoners’ health, incarceration, and re-entry are not separate problems. The health of current and former prisoners may be related to the allocation of health care organizations and the prevalence of neighborhood issues, such as coercive mobility. In combination, high rates of incarceration and re-entry produce coercive mobility (Rose and Clear, 1998) or forced migration (Thomas, 2006). High crime neighborhoods also tend to see high levels of incarceration. Together, re-entry and incarceration represent the in and out flow of residents in a neighborhood due to contact with the criminal justice system. More importantly, the phenomena contributing to coercive mobility—incarceration and re-entry—are nested in larger spatial and neighborhood phenomena. Spatially, incarceration and re-entry are both clustered. High crime neighborhoods tend to see high levels of arrest and, thus, high levels of incarceration; for instance, in New York City, there are several blocks where over a million dollars is spent annually to police and incarcerate individuals (Cadora, 2014). Similarly, returning prisoners cluster in a disadvantaged neighborhoods (La Vigne et al., 2003a; b; c; Visher and Farrell, 2005). Neighborhoods with coercive mobility^a^ typically experience other neighborhood maladies, such as poverty, high rates of unemployment, crime, residential instability, family disruption, and low social cohesion (Harding et al., 2013; Krivo and Peterson, 1996; Rose and Clear, 1998; Sampson et al., 1997). The mass reentry of prisoners to disadvantaged neighborhoods likely means that the health issues facing former prisoners and communities are spatially concentrated in a small number of neighborhoods. The resource deprived neighborhoods described above are generally ill-equipped to address ex-prisoners’ health needs (Kubrin and Stewart, 2006; Visher and Farrell, 2005).

Taken together, the facts beg the question: when coercive mobility is high, do neighborhoods have the health care organizations needed to service those individuals cycling in and out of prison? Given the durable relationship between coercive mobility, neighborhood disadvantage, and health issues (Thomas, 2006), there are several reasons why coercive mobility may be associated with the number of health care organizations (HCOs) in a neighborhood. On one hand, coercive mobility signals a strong need for health care organizations due to the poor health of former prisoners. On the other hand, because neighborhoods with high levels of coercive mobility are also disadvantaged in other ways (Rose and Clear, 1998), these neighborhood are not likely to have a sufficient number of health care organizations. While coercive mobility may be a strong indicator of need, it is unlikely that these neighborhoods are able to attract or maintain health care organizations in a rural, suburban, or urban neighborhood (Burton et al., 2013; Murphy and Wallace, 2010; Small and McDermott, 2006; Wallace and Papachristos, 2014). These neighborhoods also have a difficult time attracting health care organizations health care organizations because local patrons are unlikely to be able to afford their services (Bane, 1991; Kirby and Kaneda, 2005). As noted above, neighborhoods that experience extensive numbers of individuals churning in and out of prison are also likely to be socially disorganized and poor (Rose and Clear, 1998). These neighborhoods tend to be without health care resources despite their resident’s increased need (Kirby and Kaneda, 2005).

The spatial and neighborhood issues surrounding the placement of health care organizations are similar to those associated with coercive mobility. Researchers have long noted that there is spatial inequality in the distribution of health care organizations (Barnett, 1978; Guagliardo, 2004; Guagliardo et al., 2004). For instance, the socioeconomic status of neighborhood residents is negatively correlated with attracting doctor’s offices (Bane, 1991; Kirby and Kaneda, 2005). Additionally, spatial issues surrounding the allocation of health care organizations include the uneven distribution of providers and consumers, and issues of travel time to health care facilities (Wang and Luo, 2005). Compared to non-rural areas, rural areas are medically underserved (Frenzen, 1991; Wright et al., 1996); this is generally caused by large distances to care facilities and the cost of such travel (Bane, 1991).

The present study examines whether coercive mobility is associated with higher numbers of health care organizations in neighborhoods. To do this, we employ incarceration and re-entry data from Justice Atlas—a public, online depository for corrections data—for the state of Arkansas, as well as the 2008 County Business Patterns (CBP) data which documents organizations nationally. Using a logit-hurdle model, we estimate the both the likelihood of a neighborhood having any health care organizations and the number of health care organizations within a neighborhood. We hypothesize that higher rates of coercive mobility (i.e., incarceration and re-entry) will be associated with both lower numbers of health care organizations and having no health care organizations within neighborhoods, net of neighborhood controls.

There is no understating the importance of health care organizations for neighborhoods with high rates of coercive mobility. Not only is post-release health care associated with better maintenance of health, it is also associated with lower rates of recidivism (Dixon et al., 1993; Farley et al., 2000; Flanigan et al., 1996; Kim et al., 1997; Vigilante et al., 1999). Furthermore, recent work shows that disadvantaged neighborhoods with decreasing numbers of health care organizations have greater rates of ex-prisoner recidivism (Wallace and Papachristos, 2014). Thus, local health care organizations have the potential to lower overall crime and recidivism rates, as well as diminish the negative impact of returning prisoners’ health on neighborhoods.

## Methods

### Setting

Our study takes place in the state of Arkansas. Disadvantage can produce health disparities in rural and urban communities alike (Geronimus et al., 2006; Hattery and Smith, 2007), however, where this study takes place—the rural state of Arkansas—there is particular disadvantage in regards to residents’ health and availability of services. According to the University of Arkansas’ Division of Agriculture’s *2005 Rural Profile of Arkansas*, the health of Arkansas citizens is worse than the national average. For example, the average national five-year infant mortality rate is seven per 1,000 births, while Arkansas averages 8.8 deaths per 1,000 births. The University of Arkansas’ *2005 Rural Profile of Arkansas* also demonstrated that the Mississippi Delta and St. Francis County, Arkansas areas suffer from even higher rates of infant mortality, which during the same period were 10.5 and 12.1 deaths per 1,000, respectively. When it comes to primary care physicians, Arkansas lags behind the national average with only 83 primary care physicians per 100,000 persons; rural areas have only 71, and the Delta, only 59 (University of Arkansas, 2005).

### Data sources

To assess the relationship between health services and incarceration and release, we employ three data sources. First, our health care organizations data originates from the 2008 County Business Patterns (CBP) data. The CBP data is a count of every business or organization nationally that keeps a formal payroll (Murphy and Wallace, 2010; Small and McDermott, 2006)^b^. Each business is classified by the North American Classification System (NAICS); the NAICS codes designate business or organization type which enables differentiating between the three types of health services we investigate: all health care organizations, doctors’ offices, and mental health care organizations. We aggregate the total number of each type of health service to obtain a total count of the three health service types within each zip code in Arkansas. While this data is available longitudinally, we use the year 2008 to correspond with our coercive mobility data, which is discussed next. Zip codes are the neighborhood unit. The smallest spatial unit in the CBP data is zip code, and while zip code is an imperfect proxy for neighborhood, it allows us to approximate intra-county variation in access to resources and other important neighborhood economic forces (Murphy and Wallace, 2010; Small and McDermott, 2006).

Our second data source is from Justice Atlas, which is run by the Justice Mapping Center, a depository for corrections data for various states in the US. JusticeAtlas.org is “an online tool for mapping the residential distribution of people involved in the criminal justice system”. It uses aggregated address data to map the flow of people being removed to prison, reentering communities from prison, and the standing population concentrations of people under parole or probation supervision” (www.justiceatlas.org). The data available is from the year 2008. While the Justice Atlas data contains all zip codes in Arkansas counties, it is important to note that some zip codes span across multiple counties; these zip codes appeared in the data twice. Thus, to avoid duplicate zip codes, we retained the zip code with the largest population. This avoids the problem of zip codes with small populations influencing the analysis.

To model neighborhood characteristics, we also employ data from both the 2000 and 2010 Census, as well as the 2007 to 2011 5-year estimates of the American Community Survey. Several neighborhood characteristics variables were linearly interpolated to the year 2008 to control for neighborhood dynamics related to HCO allocation in addition to coercive mobility. Table 1 displays the summary statistics for all the variables in the study.

**Table 1:** **Summary statistics**

	**Mean**	**Standard deviation**
All HCOS, 2008	8.284	1.071
Doctor's offices, 2008	7.096	0.949
Hospitals, 2008	0.161	0.025
Mental HCOs., 2008	1.027	0.117
Coercive mobility	0.012	0.043
Concentrated disadvantage	0.000	0.044
% without a high school diploma	36.566	13.960
% unemployed	3.659	9.259
% rural	83.333	30.325
% population 65 and over	22.060	6.279
Population density	1.794	7.883
Spatial lag of all HCOs	20.536	34.147
Spatial lag of doctor’s offices	17.773	30.577
Spatial lag of mental health care organizations	2.340	2.878

### Dependent variable

In this study, we investigate the effect of coercive mobility on the availability of health care organizations. As such, there are three dependent variables: total number of health care organizations, the number of doctor’s offices, and the number of mental health care organizations. The number of health care organizations (HCOs), is a count of the number of doctor’s offices, hospitals, or mental health care organizations in each zip code. For a full listing of the businesses and organizations that consist of these variables, see Table 2.

**Table 2:** **Descriptions of the organizations/businesses that constitute doctor’s offices, hospitals, and mental health facilities**

***Doctors’ offices***		**Mean**	**Median**
621111	Doctors of osteopathy and medical doctors' offices (except mental health)	9.42	3
621112	Doctors of osteopathy and medical doctors' mental health offices	1.84	1
621210	Doctors of dental surgery and doctors of dental medicine offices	5.71	2
621310	Doctors of chiropractic offices	2.58	2
621320	Doctors of optometry offices	2.22	2
621330	Doctors of psychology offices	2.14	1
621340	Offices of physical, occupational and speech therapists, and audiologists	3.16	2
621391	Doctors of podiatry offices	1.65	1
621399	Offices of all other miscellaneous health practitioners	1.55	1
***Hospitals***			
622110	General medical and surgical hospitals	1.13	1
622210	Psychiatric and substance abuse hospitals	1.00	1
623220	Residential mental health and substance abuse facilities	1.42	1
***Mental health services & facilities***			
621112	Doctors of osteopathy and medical doctors' mental health offices	1.84	1
621330	Offices of mental health practitioners (except physicians)	2.14	1
621420	Outpatient mental health and substance abuse centers	1.60	1
622210	Psychiatric and substance abuse hospitals	1.00	1
623220	Residential mental health and substance abuse facilities	1.42	1

### Independent variable

Our independent variable, coercive mobility, is principle component factor score of the incarceration rate, the probationer and parolee release rates for each zip code in 2008. First, the incarceration rate is the “Number of People Admitted to Prison per 1000 Adult Residents (2008)” within a particular zip code (justiceatlas.org). The parolee release rate is the “Number of People on Parole per 1000 Adult Residents, 1-Day Snapshot, 2008” (justiceatlas.org), while the probationer release rate is the “Number of People on Probation per 1000 Adult Residents, 1-Day Snapshot, 2008” (justiceatlas.org). These three rates are highly correlated, but when taken collectively, they capture the churning of residents in and out of a neighborhood through criminal justice activity. As such, we collapsed the rates into a principle component factor score named “coercive mobility”. Coercive Mobility has an eigenvalue of 2.04 and no other factors were found. Factor loadings for this variables ranged from 0.797 to 0.851.

### Controls

We include several controls in our models. First, we include the *percent of the population in the zip code without a high school diploma. Percent Unemployed* is determined by creating a ratio of the number of people in the work force but who are unemployed and the total number of people in the work force in a zip code. Our study site, Arkansas, is mostly rural. Given that rural, suburban, urban areas vary in regard to access to care and other resources (Burton et al., 2013; Murphy and Wallace, 2010), we control for the *percent of the population in the zip code that are in rural areas*. Additionally, we control for *the percentage of the neighborhood population who are over 65 years old* given that older populations are associated with higher medical service needs (Auchincloss et al., 2001). Also included is *Population density* or the number of individuals per mile in the zip code. Lastly, we created a variable for *concentrated disadvantage* which includes: percent female headed households with children, percent Black, percent in poverty, and percent renter. Concentrated disadvantage is an important control for health care organization placement given that several studies have noted that the prevalence of organizational resources in neighborhoods is dependent on whether that neighborhood has the capacity to garner those resources (Burton et al., 2013; Murphy and Wallace, 2010; Small and McDermott, 2006; Wallace, 2015; Wallace and Papachristos, 2014). Concentrated disadvantage has an Eigenvalue of 2.43. All of control variables that employ census data were linearly interpolated to 2008 values and are standardized for ease of interpretation.

Next, with the exception of the model where the total number of health care organizations, we include the number of hospitals in each models. Hospitals are highly likely to drive the number of health care organizations in a neighborhood (Guagliardo, 2004; Norton and Staiger, 1994)^c^. Therefore, to account for this influence, we include the number of hospitals in a neighborhood as a control.

We also include several spatial lags to control for the impact that health care organizations in the surrounding neighborhoods have on the number of health care organizations in the focal neighborhood. Each dependent variable (all HCOs, doctor’s offices, and mental health care organizations) has a spatial lag. The average size of a zip code is just over 32 miles (Grubesic and Matisziw, 2006); therefore we calculated a distance decay effect of 35 miles. To create the spatial lags, a weight matrix (*W*) was created and then row standardized. The dependent variables and number of hospitals were then multiplied by this *W* matrix to create spatially lagged measures.

### Analysis plan

The outcomes of total number of health care organizations, doctor’s offices, and mental health care organizations are highly skewed and contain a large number of zeros. Consequently, we employ a Poisson-Logit hurdle regression model to appropriately model both the zeros and counts within our dependent variables. Similar to zero-inflated models, hurdle models are two part models which estimate both a binomial probability and a count model (McDowell, 2003). Rather than assuming that there is a group of cases that is never at risk of experiencing the outcome (here, it would be that neighborhoods could never obtain a HCO) as zero-inflated models do, hurdle models instead assume that all units of the sample are at-risk for the outcome event (Bandyopadhyay et al., 2011; Loeys et al., 2012). Thus, hurdle models are both a binary probability model (logit) and a truncated-at-zero count model (Poisson).

For the logit model, we predict the likelihood of having any level of health care organizations within a neighborhood, with 0 being no HCOs and 1 being having *at least one* HCO*.* The Poisson model assumes that neighborhoods have crossed the “hurdle” and obtained a HCO; as such, the zero-truncated count model focuses on those neighborhoods with non-zero counts of HCOs and models the number of HCOs conditional upon obtaining at least one HCO. In other words, the process only begins generating positive counts of HCOs after crossing a zero barrier or hurdle (Hilbe, 2011). By way of an example, one of our dependent variables is the number of doctor’s offices within a neighborhood. A two-part hurdle model would first estimate the binary outcome represented by 0 (i.e., no doctor’s offices) and 1 (i.e., *any* number of doctors’ offices). In the binomial models, we include coercive mobility, percent of the population in the zip code without a high school diploma, percent unemployed, the percent of the population in rural areas, the percent of the population who are over 65 years old, population density, concentrated disadvantage, and the spatial lag of the dependent variable. The second part of the hurdle model, or the Poisson, would model the outcome as counts, or the number of doctor’s office, zero not included. Here, we include the same variables as the Logit models. This type of model allows us to gain an understanding of whether coercive mobility impacts the ability of a neighborhood to gain *any* HCOs and if it impacts the ability of a neighborhood to gain additional HCOs once they have an HCO. Health service availability may also be dependent on larger economic forces related to the county. As such, we include county fixed effects.

Finally, it is important to note potential issues of multicollinearity. Table 3, which is the correlation matrix of all variables, shows that there are some relatively high correlations between variables that are unrelated (there are, understandably, higher correlations among variables that are related, like the total number of health care organizations and the number of doctor’s offices). However, the VIFs for our models are low, with an average VIF being around 2. As such, multicollinearity is not a large problem in the models.

**Table 3:** **Correlation matrix of all variables**

	**All HCOs**	**Doctor's offices**	**Mental health care organizations**	**Hospitals**	**Coercive mobility**	**Concentrated disadvantage**	**% Without a high school diploma**	**% unemployed**	**% rural**	**% population 65 and over**	**Population density**	**Spatial lag of all HCOs**	**Spatial lag of doctor’s offices**	**Spatial lag of mental health care organizations**
All HCOs	1													
Doctor's offices	0.999*	1												
Mental health care organizations	0.922*	0.903*	1											
Hospitals	0.569*	0.555*	0.486*	1										
Coercive mobility	0.128*	0.123*	0.159*	0.067	1									
Concentrated disadvantage	0.157*	0.154*	0.175*	0.033	0.171*	1								
% without a high school diploma	-0.275*	-0.272*	-0.250*	-0.270*	-0.095*	0.239*	1							
% unemployed	-0.003	-0.007	0.024	0.011	-0.025	0.206*	0.136*	1						
% rural	-0.617*	-0.605*	-0.631*	-0.467*	-0.222*	-0.335*	0.312*	-0.018	1					
% population 65 and over	-0.165	-0.161*	-0.187*	-0.091*	-0.174*	-0.296*	0.095*	0.008	0.309*	1				
Population density	0.121*	0.123*	0.085	0.145*	-0.072	0.050	-0.109*	0.023	-0.253*	-0.134*	1			
Spatial lag of all HCOs	0.335*	0.338*	0.238*	0.386*	0.118*	0.065	-0.375*	-0.031	-0.541*	-0.288*	0.506*	1		
Spatial lag of doctor’s offices	0.333*	0.336*	0.235*	0.385*	0.118*	0.069	-0.371*	-0.029	-0.539*	-0.286*	0.508*	1.000*	1	
Spatial lag of mental health care organizations	0.333*	0.335*	0.250*	0.373*	0.120*	0.020	-0.401*	-0.056	-0.546*	-0.313*	0.480*	0.972*	0.967*	1

## Results and discussion

Table 4 displays the results from the models predicting the availability of our three health care organization (HCO) outcomes. We begin with the all HCO model, Model 1. For the logit portion of the model, coercive mobility is a significant positive predictor of whether a neighborhood as at least one HCO of any type: for every one standard deviation increase in coercive mobility, the odds of having as at least one HCO of any type increases by 2.2 (e^0.776*1^). Thus, net of other neighborhood conditions, coercive mobility is a powerful predictor of HCO placement; indeed, outside of percent rural, it is the largest predictor of all HCOs. Also, there are several significant predictors of whether a neighborhood as at least one HCO of any type. The percent without a high school diploma, percent rural, and population density are all negatively related to having as at least one HCO of any type, while the percent older population is positively related to having as at least one HCO of any type. The spatial lag of all HCOs is both positive and significant, showing that HCOs from neighboring zip codes impact the likelihood of the focal neighborhood having at least one HCO of any time. Next in Model 1 is the Poisson model, which predicts the number of HCOs in a neighborhood once the neighborhood has passed the “hurdle” of having one HCO. Here, the percent without a high school diploma and the percent rural have significant, negative relationships with the number of HCOs. Coercive mobility is marginally significant at p < 0.10. Lastly, the spatial lag of all HCOs is significant, and negative; however, the coefficient size is small.

**Table 4:** **Logit-Poisson hurdle models predicting the number of health care organizations by type in 2008**

	**Health care organizations**		**Doctor’s offices**		**Mental health care organization**	
	**Logit**	**Poisson**	**Logit**	**Poisson**	**Logit**	**Poisson**
Hospitals in 2008			2.898**	0.292**	0.959+	0.155**
			(1.096)	(0.044)	(0.548)	(0.048)
Coercive mobility	0.776**	0.325+	0.713**	0.415**	0.679**	0.596**
	(0.134)	(0.191)	(0.131)	(0.158)	(0.160)	(0.189)
Concentrated disadvantage	0.197	-0.035	0.200	0.000	0.064	-0.195*
	(0.147)	(0.094)	(0.140)	(0.082)	(0.140)	(0.078)
Percent without a high school diploma	-0.360**	-0.538**	-0.379**	-0.417**	-0.332+	-0.439**
	(0.118)	(0.135)	(0.119)	(0.152)	(0.170)	(0.126)
Percent unemployed	-0.161	0.011	-0.107	-0.144	-0.231	0.139+
	(0.128)	(0.147)	(0.123)	(0.088)	(0.145)	(0.080)
Percent rural	-2.142**	-0.886**	-1.866**	-0.861**	-1.833**	-0.551**
	(0.294)	(0.091)	(0.277)	(0.082)	(0.193)	(0.101)
Percent of population over 65	0.385**	-0.009	0.361**	-0.013	0.043	-0.003
	(0.132)	(0.069)	(0.130)	(0.072)	(0.229)	(0.093)
Population density	-0.823**	0.000	-0.779**	0.037	-0.075	-0.045
	(0.157)	(0.059)	(0.149)	(0.041)	(0.134)	(0.048)
Spatial lag of all HCOs	0.039**	-0.005**				
	(0.009)	(0.002)				
Spatial lag of doctor’s offices			0.045**	-0.007**		
			(0.010)	(0.002)		
Spatial lag of mental health care organizations					-0.139**	-0.078**
					(0.048)	(0.024)
Constant	-0.583**	1.623**	-0.821**	1.336**	-1.301**	0.299
	(0.177)	(0.113)	(0.180)	(0.132)	(0.163)	(0.189)
Observations	518	518	518	518	518	518

Robust standard errors in parentheses; **p<0.01, *p<0.05, ^+^p<0.10

Model 2 predicts doctor’s offices. Note that this model has an additional control: the number of hospitals in the neighborhood. The logit portion of the model shows that coercive mobility has a significant, positive impact on whether the neighborhood has at least one doctor’s office: as coercive mobility increases above the average (0), the odds of having as at least one doctor’s office increases by 2.04 (e^0.713*1^). Significant negative predictors of the likelihood of having at least one doctor’s office include the percent without a high school diploma, percent rural, and population density. Significant positive predictors the likelihood of having at least one doctor’s office include the number of hospitals and the percent older population. The spatial lag of doctor’s offices is also positive and significant. For the Poisson portion of Model 2, coercive mobility is again positive and significant, showing that as coercive mobility increases, so does the number of doctor’s offices in a neighborhood, net of controls. Significant negative predictors of the number of doctor’s office include the percent without a high school diploma and percent rural. The spatial lag of doctor’s offices is significant and negative: as the number of doctor’s offices in the surrounding neighborhoods increase, the number of doctor’s offices in the focal neighborhood decreases.

The final model, Model 3, predicts mental health care organizations. It appears that there is a different set of predictors for mental health care organizations than for doctor’s offices or all health care organizations. In the logit portion of the model, coercive mobility significantly predicts the likelihood of a neighborhood having at least one mental health care organization: for every one standard deviation increase in coercive mobility, the odds of having as at least one mental health care organization in a neighborhood increases by 1.97 (e^0.679*1^). Only the percent rural in a neighborhood significantly predicts the likelihood of having at least one mental health care organization: for everyone one unit increase in the percent rural, the odds of having least one mental health care organization in the neighborhood decrease by 0.16 (e^-1.833*1^), or in another words, there is a 84% reduction in odds (1-0.16). The Poisson portion of Model 3 has several more significant predictors than the logit portion. First, coercive mobility is significant and positive, showing that as coercive mobility increases, so does the number of mental health care organizations in a neighborhood. The number of hospitals in the neighborhood also has a positive impact on the number of mental health care organizations, demonstrating that hospitals can facilitate the placement of other health care organizations. Concentrated disadvantage is significant for the first time in any model: here, higher levels of concentrated disadvantage are associated with lower numbers of mental health care organizations. The percent without a high school diploma and percent rural are also significant and negatively associated with the number of mental health care organizations in a neighborhood. Lastly, the spatial lag for the number of mental health care organizations is significant and negative.

In sum, coercive mobility is associated with both the higher likelihood of having at least one HCO and the number of HCOs in neighborhoods for all three outcomes (note though that for all health care organizations, the significance level was p<0.1). Given the relationship that coercive mobility has with detrimental neighborhood outcomes, like concentrated disadvantage and lower levels of education, the positive relationship coercive mobility has with health care organizations is surprising. In the coming section, we discuss the implications of these findings.

## Conclusion

Prisoners are likely to be unhealthy when they enter prison, have the potential to see their health degrade while incarcerated (Wakefield and Uggen, 2010), and when they are released into the community, their health problems follow them. In this project, we look to whether the neighborhoods seeing individuals churn in and out of prison are stocked with the health care organizations these individuals will likely need to gain positive post-release outcomes like good health, substance abuse or mental health treatment, and criminal desistance. We find a two pronged relationship between coercive mobility and the presence and number of health care organizations. We turn to these results next.

First, we find that as levels of coercive mobility increase in a neighborhood, the likelihood that the neighborhood has one or more health care resources also increases. Additionally, coercive mobility positively predicts the number of doctor’s offices and mental health care organizations in a neighborhood. Both of these findings are counterintuitive to literature suggesting disadvantaged neighborhoods, such as those with high levels of coercive mobility, have difficulty gaining access to and keeping health care organizations (Kirby and Kaneda, 2005; Murphy and Wallace, 2010; Wallace, 2015; Wallace and Papachristos, 2014). Indeed, concentrated disadvantage was only significant in predicting the number of mental health care organizations; here there was a negative relationship. Thus, it appears that coercive mobility does signal some level of need for health care organization in a neighborhood that is above and beyond the need signaled by other neighborhood ills like concentrated disadvantage.

As such, our study does generates a positive conclusion—that neighborhoods in need of health care organizations due to the population of prisoners cycling in and out of prison—are more likely to have those organizations. This is important for four reasons. First, public health research shows that individuals living closer to health care organizations are more likely to report better health and other positive health outcomes (Cohen et al., 2006; Crowder and Teachman, 2004; Entwisle, 2007). This is of outmost importance for current and former prisoners given that they exit prison with few personal resources, like transportation (Petersilia, 2003). Next, research has demonstrated that adolescents in poor health are more likely to be deviant than their healthier counterparts (Jones and Lollar, 2008; Suris and Parera, 2005). When applying this to individuals cycling in and out of prison, keeping them healthy may lower the likelihood of committing future crime. Indeed, research demonstrates that this is the case for specific types of criminals, like substance users or HIV positive individuals (Dixon et al., 1993; Farley et al., 2000; Flanigan et al., 1996; Gaes et al., 1999; Kim et al., 1997; Vigilante et al., 1999; Visher and Courtney, 2007; Wexler et al., 1999). Lastly, as the number of individuals in the neighborhood having contact with the criminal justice system grows there is a higher likelihood that those individuals are spreading infectious diseases and straining health care organizations currently in place (Hipp et al., 2010). The association of coercive mobility with higher numbers of health care organizations in neighborhoods make enable communities to better cope with infectious disease. And lastly, access to health care organizations likely helps ex-prisoners meet their parole and probation conditions. Health conditions like substance abuse and mental health problems tend to be tied to conditions of parole and probation. Therefore, the prevalence of health care organizations is often tied to recidivism (see Wallace, 2015; Wallace and Papachristos, 2014). Thus, health care organizations potentially play an important role in keeping residents and offenders alike healthy, and potentially, lowering neighborhood crime and recidivism rates.

There are several limitations to our study worth noting. We are unable to capture trends in our data. While we have longitudinal data for the healthcare organizations, the data on coercive mobility is not available over time. Future work needs to track these trends longitudinally to see how they influence each other. Next having zip codes as the neighborhood unit is not ideal. Researchers have suggested that zip codes are too large to look at specific neighborhood level outcomes, yet others suggest that zip codes are ideal for examining large-scale demographic and economic processes (Small and McDermott, 2006). Ideally future research would examine coercive mobility and health care organizations at multiple levels, including at a smaller neighborhood unit such as the census tract or neighborhood block group. Also, the zip codes in Arkansas that border other states are likely influenced by their neighbors in those states. While we are able to acquire census and organizational data at a national level, information regarding the parolee rate, probation rate, and prison admissions rate are not available nationally from Justice Atlas. Future research should also account for this issue. Lastly, while we note how hospitals drive of the placement of other health care organizations, pertinent information in establishing this relationship, such as who owns the hospital and HCOs, their affiliations, and specialty, is not available in the NAICs data. Future research would do well by accounting for this when looking at how criminal justice activity in a neighborhood impacts HCO availability.

In conclusion, future public policies should aim to locate health care organizations in areas where there is significant churning in and out of prison. Given that this population is likely to have a strong impact on the health of the local population, having significant numbers of HCOs may keep offenders from continuing to pass through prison, and potential lower neighborhood crime rates as well.

## Endnote

^a^Although coercive mobility is related to detrimental neighborhood conditions, coercive mobility is distinct from other neighborhood conditions. Returning prisoners are subject to intense monitoring (e.g., parole and probation), policing, and the involvement of corrections agents in their neighborhoods (Fagan et al., 2002). As such, coercive mobility, while certainly related to other neighborhood characteristics, is generated through a process distinct from social disorganization or concentrated disadvantage. Indeed, recent scholarship demonstrates that while reentry is associated with social disorganization and concentrated disadvantage, neighborhood level reentry and incarceration exert *independent* influences on criminal justice outcomes, which recidivism (see Chamberlain and Wallace, 2015). This early evidence suggests that coercive mobility is independent from these neighborhood effects and may also be independently related to how health care organizations are allocated. Furthermore, as our results will show, coercive mobility and concentrated disadvantage have opposing effect on the allocation of HCOs. Thus, they seems to work independently on the outcome.

^b^While generally considered accurate, the Census reports some issues with non-response when gathering information about small businesses. Here, item non-response is more of an issue than general non-response because all business are required to answer the survey (see http://www.census.gov/econ/census/help/methodology_disclosure/data_processing_and_treatment_of_nonresponse.html for more information). When this occurs, the Census imputes the missing data, which consists of only 5% of the final data reported by the Census for the CBP data. Lastly, item non-response is less likely to be an issue in this analysis since the study does not model the characteristics of the organization and is only concerned with the presence of the organization in a neighborhood.

^c^The size of the hospital also influences the availability of HCOs given that larger hospitals are more likely to bring other HCOs in to the neighborhood. Unfortunately, there are limitations to the data in regards to additional specification surrounding health care organizations. The data does enable us to differentiate between hospital sizes via the number of employees. We differentiate hospitals by size using the following categories: small hospitals have between 1 and 20 employees, medium hospitals have between 20 and 100 employees, and large hospitals have over 100 employees. The average number of small hospitals in a neighborhood is 0.57 (sd = 1.11), while the average for medium sized hospitals is 0.14 (sd = 0.43), with large hospitals having an average of 0.22 (sd = 0.53). Thus, most of the hospitals in Arkansas appear to be smaller sized hospitals. Given the low numbers of larger and mid-sized hospitals in the data, we were not able to ascertain their effect on the allocation of resources. As such, we simply used an overall count of hospitals in our models.
